# Catastrophic case of the total knee arthroplasty dislocation: A case report

**DOI:** 10.1016/j.ijscr.2024.109925

**Published:** 2024-06-21

**Authors:** Mohammad Mozaffar, Mehrdad Sadighi, Amir Sabaghzadeh, Farsad Biglari, Meisam Jafari Kafiabadi, Amirhossein Zolghadr

**Affiliations:** a Department of General and Vascular Surgery, Shohada Tajrish Medical Center, Shahid Beheshti University of Medical Sciences, Tehran, Iran; b Department of Orthopedic Surgery, Clinical Research Development Unit of Shohada-e Tajrish Hospital, Shahid Beheshti University of Medical Sciences, Tehran, Iran; c Physiotherapy Research Center, Shahid Beheshti University of Medical Sciences, Tehran, Iran; d School of Medicine, Tehran University of Medical Sciences, Tehran, Iran

**Keywords:** Artery, Ischemia, Joint, Total knee arthroplasty, Vascular injury

## Abstract

**Introduction and importance:**

Arterial injury is extremely rare after total knee arthroplasty.

**Case presentation:**

We describe a 68-year-old woman with dislocation of total knee arthroplasty after falling from a height. She had a popliteal artery injury and a vascular bypass was performed in delay. The patient died of a second myocardial infarction 3.5 months after her first introduction to our center.

**Clinical discussion:**

Due to the prominent risk of vascular injuries after dislocation in TKA patients, we recommend performing vascular evaluations using CT angiography for all patients.

**Conclusion:**

Any untreated vascular compromise in the setting of TKA dislocation may lead to devastating outcomes such as amputation and death.

## Highlights

• Knee dislocation is extremely rare after total knee arthroplasty.
• Highly recommended to perform vascular evaluation by CT angiography after dislocation in TKA patients.
• Any untreated vascular compromise in the setting of TKA dislocation may lead to devastating outcomes such as amputation and death.

## 1 Introduction

Arterial injury is extremely rare after total knee arthroplasty (TKA) [1]. Knee dislocation can result in catastrophic outcomes such as injury to the popliteal artery or nerve, leading to amputation. Therefore timely diagnosis and proper management of this condition are crucial [2,3]. To achieve this goal, the frequency of clinical examinations and the sensitivity of vascular tests, such as CT angiography, should be enhanced to avoid misdiagnosis and further limb amputation.

Several risk factors for knee dislocation after TKA include trauma, polyethylene wear, extensor mechanism disruption, component malposition, extension-flexion gap mismatch, soft-tissue imbalance, and inappropriate selection of the primary implant [4]. Hyperextension or other unrestrained motions rupture the posterior capsule, followed by anterior dislocation. In this way, the content of the popliteal fossa such as the popliteal artery would be damaged [1].

## 2 Case presentation

In this study, we report a case of knee joint replacement dislocation and the work has been reported in line with the SCARE criteria. Total knee arthroplasty (TKA) surgery with posterior stabilized (PS) design of the left knee was performed on a 68-year-old woman and she was discharged without any early postoperative complications and good health status. Eight months later, the patient developed an anterior knee dislocation after falling from a height. She underwent the close reduction (CR) in another center and was immediately discharged from the hospital with a knee brace. Five days after CR the patient complained of calf pain and hypothermia in the reduced limb. After examination by an orthopedic surgeon, she was referred to our tertiary trauma center with the impression of vascular detrition.

**Fig. 1:**
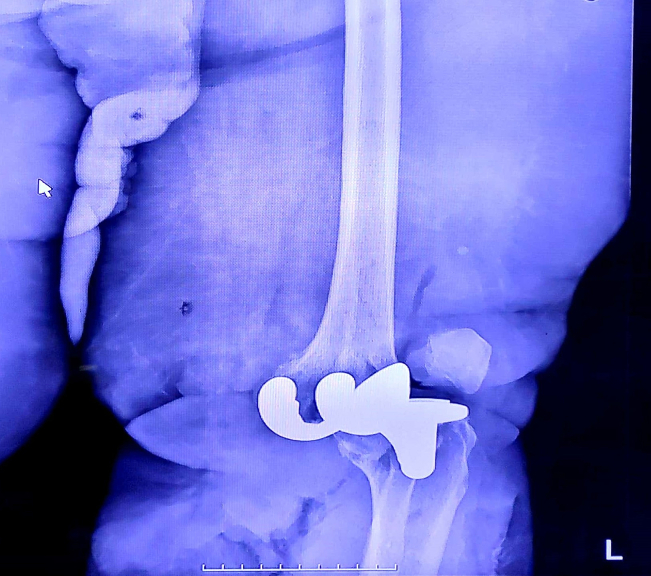
Lateral plain radiograph of the left knee.

**Fig. 2:**
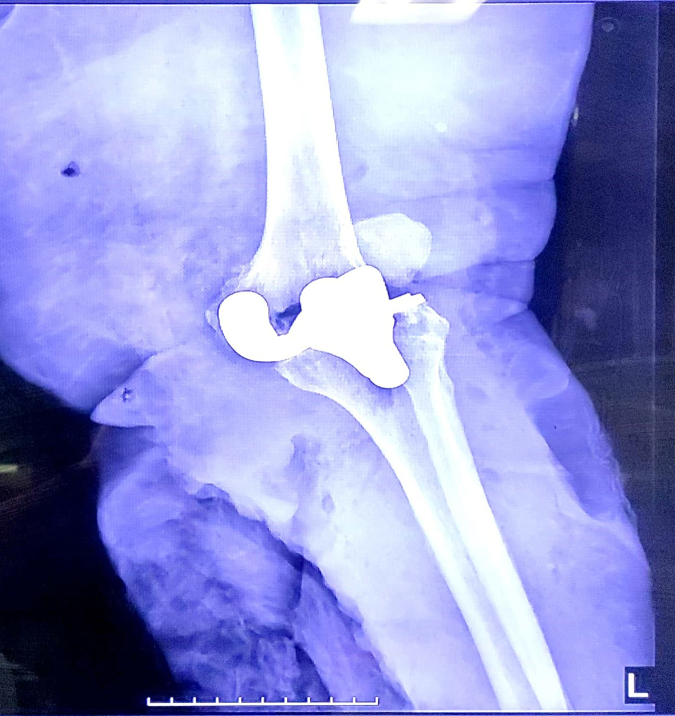
Anteroposterior plain radiograph of the left knee.

On clinical examination the left leg was cold from mid-calf down, the distal capillary filling was impaired, and dorsalis pedis and posterior tibialis artery pulses were not palpable. Sensory and motor deficit of the leg was detected. Radiography showed dislocation of the TKA (Fig. 1, Fig. 2).

**Fig. 3:**
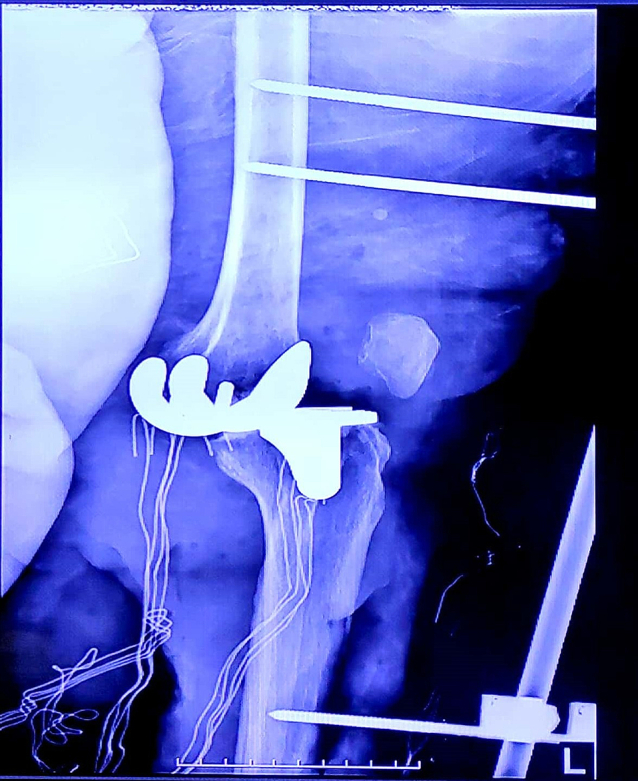
Lateral plain radiograph of the left knee after external fixator.

CT angiography revealed a cut-off at the popliteal artery and distal runoff was seen. The vascular surgeon admitted the patient to the operating room with the impression of acute ischemia and thrombosis due to arterial intimal injury. To save the limb, a vascular bypass using the reverse saphenous vein graft (RSVG) method was conducted, spanning 20 cm from the high to low popliteal part of the knee. To reduce the pressure of leg compartments, classic fasciotomy with medial and lateral incisions was done in the same surgery. Since the viability of the muscular component was questionable and the long ischemic time external fixation was performed for the patient without reducing the dislocated joint (Fig. 3). After the operation distal pulses were detectable and the patient reported pain relief.

At first look deep compartment's muscles had a normal state, though superficial posterior, anterior, and lateral compartments were ischemic and muscles did not respond to electrical stimulation. During hospitalization irrigation and debridement (I&D) was regularly done to prevent infection.

**Fig. 4:**
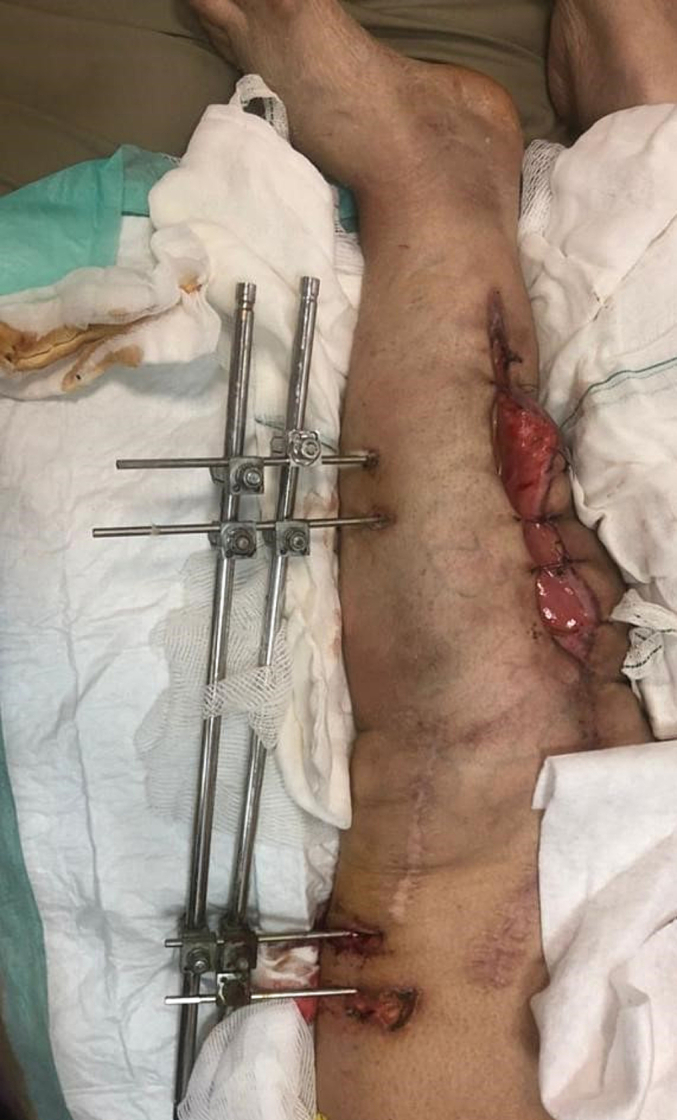
Wound condition of the leg.

On second look because of the muscle necrosis, muscle autolysis occurred (Fig. 4). One month later, while the patient was still admitted to the orthopedic ward she developed chest pain, and myocardial infarction (MI) was diagnosed based on clinical and ECG. Prompt management of MI was started after consultation with an expert cardiologist.

Two weeks later, the patient developed covid-19 infection that required ICU admission and intubation and died.

## 3 Discussion

The mechanism of anterior knee dislocation is extreme hyperextension with rotational force and there is no significant difference between natural knees and TKA patients [6]. Major vascular injury is a rare but serious complication after the dislocation of TKA [7,8]. The most common vessel predisposed to injury is the popliteal artery [7,9]. Due to the rarity of vascular injury after TKA, the association between the type of prostheses and vascular injuries in cases of knee dislocation is not well defined.

There is a report of a case with a hinge total knee prosthesis that was dislocated after falling highlighting the importance of balancing the soft tissue [10]. Conti et al. reported a case of knee dislocation 10 years after successful TKA without any postoperative instability, neurovascular problem, or pain. However, one of the follow-up radiographs revealed a slight polyethylene insert wear which caused knee joint predisposition to dislocation.

In another case, a woman with dislocated TKA with CR prosthesis experienced genicular artery occlusion and peroneal nerve palsy. The dislocated knee was managed with a PS prosthesis [11]. Dislocation of TKA may occur several years after surgery. Constantinescu et al. presented a case of 70 years-old female who experienced anterior dislocation of right TKA with popliteal artery injury 22 years after the operation. Vascular injury was managed successfully by using the contralateral great saphenous vein [12].

Knee dislocation after TKA followed by vascular injury can lead to multiorgan damage. Novotny T. et al. reported a case of knee dislocation after TKA complicated by popliteal artery injury. Although the arterial transection was surgically fixed in this patient, post-operative reperfusion syndrome caused acute chronic kidney failure. Subsequently, the patient's condition led to the amputation of the limb. Three days later their patient died of multiorgan failure and cardiovascular collapse [13].

Because of the grave consequences of the TKA dislocation and the high risk of amputation, we recommended that every TKA dislocation should be considered seriously and CT angiography (as soon as possible) and close follow-up should be performed. Even after TKA dislocation if the distal pulse is palpable, minor injury of the popliteal artery is not excluded and a high index of suspicion is the goal. In most cases the closed reduction dislocated knee is not sufficient and revision surgery is required to achieve a stable joint.

## 4 Conclusion

Due to the prominent risk of vascular injuries after dislocation in TKA patients, old age, and several comorbidities, it is highly recommended to perform vascular evaluation by CT angiography in all patients. Any untreated vascular compromise in the setting of TKA dislocation may lead to devastating outcomes such as amputation and death.

## Patient informed consent

Written informed consent was obtained from the patient's relatives for publication and any accompanying images. A copy of the written consent is available for review by the Editor-in-Chief of this journal on request.

## Ethical approval

No need for approval from an ethics committee for such a type of article (case report). Written informed consent was obtained from the patient for the publication of this case report and accompanying images. A copy of the written consent is available for review by the Editor-in-Chief of this journal on request.

## Funding

No funding.

## Author contribution

Concept or design of the article and collection of data was performed by Meisam Jafari Kafiabadi and Amirhossein Zolghadr, writing the paper was with Farsad Biglari and Amir Sabaghzadeh, and consult was performed by Mohammad Mozaffar, Mehrdad Sadighi.

## Guarantor

Meisam Jafari Kafiabadi guarantor for this case report.

## Conflict of interest statement

All authors do not have conflicts of interest.
